# Link workers’ and clients’ perspectives on how social prescribing offers a social cure for loneliness

**DOI:** 10.1177/13591053241274090

**Published:** 2024-08-22

**Authors:** Shaun Hayes, Leah Sharman, Niamh McNamara, Genevieve Dingle

**Affiliations:** 1 The University of Queensland, Australia; 2 Nottingham Trent University, UK

**Keywords:** social cure approach, social identity theory, social prescribing

## Abstract

Social prescribing is a healthcare model designed to reduce loneliness and improve individuals’ health by addressing unmet social needs. The present study adopted the Social Cure framework to provide an understanding of the psychosocial processes involved in helping participants to engage with social activities, from both the link workers’ and clients’ perspectives. Semi-structured interviews were conducted with 15 link workers (*M*_age_ = 40.12; 87% female) and 15 clients (*M*_age_ = 55.33; 73% female, 7% non-binary) of social prescribing programmes across Australia and the transcripts were analysed using reflexive thematic analysis. Three overarching themes were identified: (1) Breaking Down Barriers, (2) Finding Fit with Others, and (3) Rebuilding a Sense of Self. These findings communicate how social prescribing addressed the psychosocial barriers of clients, and how joining groups that fostered positive shared social identities resulted in meaningful improvements to clients’ well-being.

Social Prescribing (SP) is a non-medical intervention that links individuals to community services, groups, and activities to address their unmet social needs, better manage their health-related behaviours, and improve their health outcomes (Global Social Prescribing Alliance, 2021). There are many models of SP, ranging from lighter approaches where a health worker signposts available groups to clients, to holistic approaches where a link worker works closely with clients and focuses on wider social determinants of health (Kimberlee, 2015). The holistic model consists of three components: (1) the individual is referred to the programme, often by a GP or allied health professional; (2) the individual meets with a link worker to discuss their interests and needs, and learn about the activities and groups within their local community; and (3) the individual engages with a meaningful social activity within their local community (Dingle and Sharman, 2022; Drinkwater et al., 2019; Kimberlee, 2015). Through this process, SP services seek to alleviate social isolation and feelings of loneliness, and correspondingly mitigate the associated increased risk of a range of physical and mental health conditions (Holt-Lunstad, 2017; Wang et al., 2017).

The effectiveness of SP has been evaluated in multiple trial studies (Aggar et al., 2021; Carnes et al., 2017; Kellezi et al., 2019; Stickley and Hui, 2012a, 2012b; Woodall et al., 2018) and systematic reviews (Chatterjee et al., 2018; Costa et al., 2021; Reinhardt et al., 2021; Thomson et al., 2015). These benefits include reduced loneliness, anxiety, and depression and increased group memberships, confidence and self-esteem, social communication, and improved indicators of physical and mental wellbeing (Aggar et al., 2021; Chatterjee et al., 2018; Halder et al., 2021; Kellezi et al., 2019). A recent meta-synthesis of studies interrogating the effects of SP on loneliness took this further, examining both the perceived benefits and drawbacks of SP (Liebmann et al., 2022). The authors found participants perceived many benefits from SP, and a desire to continue connecting with others. However, participants also described fewer benefits and desire to connect with others when they were not interested in the available activities, when their peers did not share their interests, or they felt they did not ‘fit in’ with the activity group in which they were placed.

Despite the positive findings of these studies, and perhaps due in part to the diversity of programmes offered and outcomes reported, little is known about *how* SP works. This calls for an understanding of the psychosocial processes involved in successfully engaging clients into social programmes that support their health and wellbeing. As a result, SP has been criticised by some researchers for lacking a theoretical model to guide its implementation (Frostick et al., 2019). Clearly, SP is not as simple as increasing people’s social contact. People must experience a good ‘fit’ with their new social group and develop a sense of belonging and identification with the group to experience its psychosocial benefits. One theoretical framework through which to understand these elements is the Social Identity Approach to Health (SIAH; Haslam et al., 2018; Jetten et al., 2012).

## Social prescribing as a social cure for loneliness

Otherwise known as the ‘Social Cure’ framework (Haslam et al., 2018; Jetten et al., 2012), SIAH posits that group memberships affect our health and wellbeing when they contribute to our self-concept (Tajfel and Turner, 1979; Turner et al., 1994). Shared social identity (such as ‘us members of team X’) allows group members to harness the collective esteem and psychosocial benefits of the group (Steffens et al., 2017). When this self-concept is positive, it enables a range of health promoting psychological resources such as self-esteem, control, and social support (Greenaway et al., 2015; Guan and So, 2016; Jetten et al., 2015). Conversely, this self-concept can become negative when an individual belongs to harmful, stigmatised, or incompatible groups (i.e. where the individual finds it difficult to be a member of a particular group alongside other groups in their social landscape simultaneously). Dubbed ‘Social Curse’, this can also have substantial negative impacts our health and wellbeing (Muldoon et al., 2019). According to the Social Cure framework, loneliness occurs when people experience a lack or loss of social group identities and associated psychological resources (Hayes et al., 2022). Seen through this lens, SP is predicted to reduce loneliness and increase health and wellbeing when participants are supported to join and identify with a new meaningful group that is positive and compatible with their sense of self.

Quantitative and qualitative research in the UK has provided preliminary empirical support for the Social Cure framework being a useful lens through which to understand how SP works. For example, in a Nottingham-based SP evaluation, the relationship between increased group memberships through SP and reduced primary healthcare service usage was mediated by an increased sense of community belonging and reduced loneliness (Kellezi et al., 2019). A companion paper from the same project reported that the link between increased group memberships and quality-of-life was serially mediated by belonging, support, and reduced loneliness (Wakefield et al., 2022). Other studies have applied the Social Cure framework to examine how joining arts-based groups such as community choir singing and creative writing lead to reduced loneliness and improved wellbeing over time (Dingle et al., 2013; Williams et al., 2019, 2020). Namely, to the extent that participants formed a meaningful shared identity, they experienced improved wellbeing across multiple domains.

## Facilitators and barriers to forming a shared group identity

So, what are the psychosocial processes that facilitate or hinder the development of a meaningful shared identity with a social programme as understood through the SIAH? In the mixed-methods Nottingham study, clients described how fear and anxiety around leaving home and meeting new people presented a substantial challenge to overcome, and that central to surmounting these were having positive experiences with link workers and groups that included meaningful connection, feeling supported and listened to, and fitting in with others (Kellezi et al., 2019). In another study of a choir for people with post-stroke aphasia, the group leaders’ role in facilitating positive group experiences and encouraging participants to share their experiences contributed to their sense of belonging and cohesiveness (Tarrant et al., 2018). Central to both accounts is the clients’ identification with the group, and this group identity contributing positively to their sense of self. This sense of shared identity is facilitated by clients having a predisposition to the group (e.g. from positive previous experience, membership, or interest), the group members have a lot in common with each other, and the group as a whole is distinct from others (Tarrant et al., 2020). These elements, which should allow the client to reflect on and clarify their self-concept from belonging to groups they increasingly identify with, have yet to be meaningfully examined in the context of SP.

In contrast to the facilitators of SP, few studies that have examined the barriers experienced in the context of SP. Those that have tend to focus on service level and structural challenges, such as inappropriate client referrals, lack of available/appropriate groups and activities to refer clients to, and inadequate link worker training (Wildman et al., 2019). However, little research has considered the *psychosocial barriers* that prevent clients from joining groups and feeling socially connected prior to and during their engagement with SP (Hassan et al., 2023). From a social cure perspective, the psychosocial barriers and facilitators are fundamental in determining the success or failure of the SP group referrals made by the link worker. These include failing to build trust and confidence in clients and linking clients to groups poorly suited to their life needs and preferences (Sharman et al., 2022; Stuart et al., 2022). Stuart et al. (2022) specifically noted that it was the psychosocial needs and preferences of the clients that presented the most important barriers to joining and participating in groups. Namely, that it was problematic to link clients who had accumulated a lifetime of social challenges with groups that only superficially – not meaningfully – met their psychological and social needs, and with which they did not felt they fit in with. Therefore, the aim of the present study was to qualitatively examine perspectives of both link workers and clients on how successful SP (a) helps clients address these psychosocial barriers, and (b) develop and sustain shared social identities in community-based group programmes. Understanding these psychosocial processes from a Social Cure perspective will provide knowledge on what link workers, group facilitators, and group members can do to help new members to develop a shared identity with the new group.

## Method

### Participants

#### Link workers

Link workers from 10 SP schemes around Australia were approached to participate in the study. These schemes were identified through chain referrals from other organisations and online searches. Of these link workers, 15 agreed to participate from prescribing programmes across Queensland, New South Wales and Victoria. The majority (53%) worked within primary care-based schemes, while the remainder were placed within community-based schemes. Primary care-based link workers primarily engaged with clients internally referred to the scheme by their GP at a primary care service. In contrast, community-based link workers engaged with clients referred to the team by their GP, friends, family, or the client themself (self-referral) at a community or neighbourhood centre. Most link workers were female (87%) and ranged in age from 24 to 61 years-old (*M* = 40.12). Link workers had worked in an SP role between 3 months and 7 years.

#### Clients

Clients from three SP schemes based in south-east Queensland were approached to participate in a wider SP evaluation. These clients were identified by their link workers as open to participating in research activities. Of these clients, 15 agreed to participate in the present study. The majority (80%) were enrolled within community-based schemes, while the remainder were enrolled within primary care-based schemes. Primary care-based clients were referred to community-based groups through their link worker at the primary care service they regularly attended. In contrast, community-based clients were referred to community-based groups in their local area or at their local community centre. Most participants were female (73%) followed by male (20%) and non-binary (7%) and ranged in age from 32 to 78 years-old (*M* = 55.33). Clients had participated in SP between 3 weeks and 3 years.

### Interviews and analysis

Semi-structured interviews were conducted with questions focussed on: the interviewee’s perspectives of client barriers; what strategies and processes helped address and overcome these; what approaches link workers and clients used to navigate group activities and dynamics; and the positive and negatives of the SP approach for both the link worker and client samples. Link workers were interviewed between June 2020 and November 2021, and clients between April 2021 and June 2022. Consequently, both link workers and clients experienced a range of public and organisational COVID-19 restrictions and lockdowns during these periods. Interviews were conducted over the phone or in person by qualitative researchers SH, LS, GD, DN and ST, and all interviews were recorded and transcribed verbatim. Link worker interviews averaged 30 minutes (22–39 minutes) and client interviews averaged 31 minutes (15–51 minutes) in duration.

All interviews were qualitatively analysed using reflexive thematic analysis. Reflexive thematic analysis is an iterative process that uses both experiential and constructionist approaches (Terry et al., 2017). This was chosen for the current study as the experiential approach permitted researchers to explore meaningful elements of both the link workers’ and clients’ experiences in the analysis. Alongside this, the constructionist approach allowed the researchers to ask both link workers and clients questions about aspects of their experience as guided by the research questions. The analysis followed a well-established six-phase procedure described by Braun and Clarke (2019), comprising (i) becoming familiar with the data; (ii) generating initial codes; (iii) constructing themes from these codes; (iv) reviewing these themes against the data; (v) further defining the themes; and (vi) reporting results, with co-authors commenting and providing feedback. Analysis of the data was led by SH with LS and GD reading responses and contributing to discussions of theme meanings and label refinement, and UK-based author NM providing additional written input. The researchers have backgrounds in clinical, social and health psychology.

### Ethics

Ethics approval for this research was granted by the University of Queensland Human Research Ethics Committee (2020001019). All participants provided written and/or verbal consent before the interviews. Link workers were provided $60 shopping vouchers for their participation. Clients were provided $40 shopping vouchers for their time on top of the compensation received for their participation in the wider survey.

## Results

**Figure 1.**
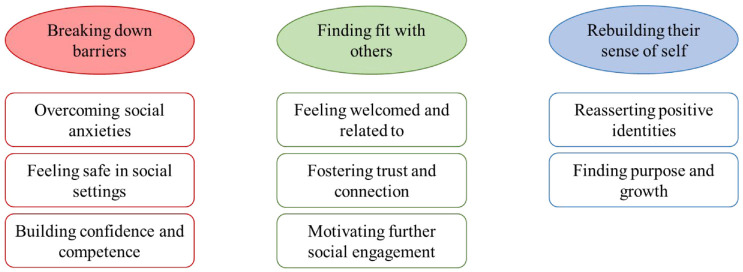
Thematic map of how SP assists clients in addressing psychosocial barriers and developing positive shared social identities.

Three overarching themes were constructed from the data and are presented below in the thematic map (Figure 1). These comprised of breaking down barriers, finding fit with others, and rebuilding a sense of self. Each overarching theme consisted of several sub-themes which further identified how successful SP assisted clients in addressing psychosocial barriers and developing positive shared social identities.

### Breaking down barriers

The first overarching theme concerned the importance of helping clients address psychosocial barriers to participation at the beginning of the SP process. Three sub-themes were identified: client’s overcoming their anxiety, feeling safe in social settings and building up their confidence and competence. Both link workers and clients highlighted the importance of addressing these barriers to create a foundation for subsequent positive social experiences.

#### Overcoming social anxieties

Anxiety was frequently mentioned by both link workers and clients as a barrier to client engagement with others. This most commonly took the form of social anxiety, which prevented clients from both initiating, building and continuing social interactions:

*Oh, I was shy. I have anxiety, fear of talking to people, fear of being involved, fear of getting into conversations with them, fear of continuing the conversation, all that kind of stuff. –* C8.

Link workers and clients also reported the role of anxiety associated with physical and mental health conditions, the disconnection resulting from these conditions and the resulting negative impact on relationships with friends and health professionals.

*I mentioned life happened at the end of my psych degree and life was happening at the end of my social work degree and I was just about ready to give up. I do have that history of mental health issues and I felt like I had nothing to give the world. I felt that I was a burden. –* C6.

Both groups of interviewees reported a range of strategies that helped in reducing these anxieties during the beginning of the SP process. Central to these were creating a space that reduced the social pressure on the client to help them feel more comfortable with the linkage process overall from the start, and the social situation at hand:

…*I was told, “Look, she won’t talk to you for the first few sessions.” …So I pretended I was running late, had to sit in the space for a little while so she could look around the room and feel comfortable, be a bit of an idiot in front of us just to lighten the space up and that sort of thing and gave her about 10 minutes and then I couldn’t shut her up.* – LW6.

#### Feeling safe in social settings

A lack of safety and the fear associated with how joining groups might lead to harm was a concern echoed among link workers and vulnerable clients. This was particularly prominent for clients with a disability or a history of domestic or sexual violence:

*… a lot of the women from [small island countries] who have come escaping domestic violence, they seem to be quite fearful of joining community groups in case they’re identified by someone in the community and they have ongoing fear that they might have retributions still.* – LW4

While link workers primarily connected these clients with organisations and services specialising in addressing these acute issues, clients reported that linking processes and groups which incorporated elements that emphasised safety, security and compassion (i.e. safe spaces, trauma-informed approaches) helped them to better engage with others and address their isolation. This was also more effective when these safety measures communicated when the client began the linking processes and before they joined their groups, which created a foundation of perceived safety for ongoing participation and engaging with new groups.

*At first it was hard. Because they want to help you, but they need to get background information. So, it was uncomfortable, but it was useful and I felt safe… I didn’t ever feel like I was pressured, like I had to - If I was uncomfortable or anything, then I could leave the room and then I’d have support if I needed to leave.* – C5.

#### Building confidence and competence

Highlighted alongside the anxiety common to clients was a lack of confidence in themselves, their social skills and their general ability at both new and old activities. Link workers especially noted the contribution of globally low self-confidence to how clients felt:

*… a lot of them were saying that they were ready to join a group, but as I was working with them, more and more excuses were being told to me from them. And what I worked out is that they didn’t actually have the confidence to go ahead.* – LW7.

This lack of confidence was also often tied to negative views of oneself and a lack of ‘practice’ in social situations and was particularly salient at the start of the SP process. However, it was also described as being distinct from the social anxiety and fear many experienced. Most participants noted that the support from the link worker followed by engaging with groups and ultimately successfully navigating social situations helped empower clients and grow their sense of confidence.

*So, a lot of people come in. And when you’ve been isolated for so long, a lot of the social skills, it can feel a bit overwhelming. So, usually what I would do is I would just sit with them and talk with them and then move them into a crowd of people, but still stay present with them. It’s incredibly miraculous to watch, because you just see them light up and they’ll just engage in conversations on their own.* – LW14.

### Finding fit with others

The second overarching theme that was constructed concerned how participants were able to fit in and positively engage with the groups they joined during SP. Three sub-themes were identified: clients feeling welcomed and related to, a greater sense of trust and social connection, and increasingly motivated to engage socially. Both link workers and clients reported that developing this sense of belonging and engagement was essential to the success of the SP process.

#### Feeling welcomed and related to

Crucial to the success of SP from both perspectives was an emphasis on groups welcoming clients and helping them feel accepted and related to when they joined. Not only did client’s report lack of this as a significant contributor to their social disconnection, but also as the most common reason for unsuccessful linkages:

*… I might clash with someone or I might be an outsider and not be welcomed in.* – C4

Although some severely struggled with this, clients who tended to be more open to SP recognised that this experience was universal to social interactions:

*And I’ve found that some just didn’t have the right fit. Some had a semi-fit, some were totally good. So, it was a total mix of things.* – C7.

Extending upon this, clients conveyed the most effective element of the linkage process was not simply linking them to group activities they were interested in, but linking them to groups where they would have shared experience and understanding with fellow members:

*Well yeah, I found the [afternoon group] that I go to are really good in the fact that they’re all around my own age and they all talk about their different experiences that they’ve had.* – C9.

#### Fostering trust and connection

Common to the accounts from both link workers and clients was a sense of mistrust in others. For many this was characterised by concerns of being exploited or mistreated, particularly by those in position of power such as health professionals (including link workers themselves). While this mistrust applied to many individuals and groups throughout the SP process, it was noted as a significant hurdle for link workers and group facilitators to manage in working with clients throughout the whole linkage process:

*Mostly, they feel themselves are very vulnerable and then sometimes they feel like they are treated unfairly by the system. So they were afraid or they were overwhelmed about experiencing the same thing again.* – LW10.

To overcome this, link workers reported dedicating substantial time building rapport with clients. Clients and link workers then utilised this sense of trust as a scaffold by which the client could extend to the group facilitators of their new groups, and their new group members thereafter. With the management strategies of their individual barriers in place and a welcoming reception, clients would be more willing to trust their new peers, connect with them meaningfully, and feel as though the new group was a good fit for them.

*… you’re creating a trusting relationship, you’re creating an empathic environment, which is a very key thing you need to do with anyone who’s in a vulnerable state. And then after that’s accomplished, the connection of them to activities that connect them to other people, that often is something that very quickly clicks in.* – LW14

#### Motivating further social engagement

Although struggles with anxiety or safety prevented many clients from accessing their community and building social connections, many others reported ongoing difficulties finding the motivation to leave their home and engage with others:

*And I have to get myself motivated to get out of this house or I’d sit here and do nothing in the cold.* – C12.

Unlike social anxiety or safety concerns which had to be addressed early in the linkage process, link workers consistently reported trying to build this motivation in clients gradually throughout the linkage process through focussing on client strengths relevant to the group activities they were interested in:

*So when we focus on what a person can do, rather than what they can’t do, the result is basically increasing their confidence and their motivation to increasing supports and the social networking.* – LW9.

While clients found the link worker’s focus on their strengths and their newfound confidence as motivating, they noted that having the support of others – most particularly peers in their new groups – and enjoyment of the group itself as the biggest driver of improvement. As this motivation continued to improve and clients felt more part of the group, many became more proactive in their social interactions beyond the group’s activities and sessions:

*… I’ll just go out and I’ll pick them up and we’ll go and have coffee. Because some of them are a lot older ladies than I am and they can’t get out. So I often go and pick some of them up and we go and have coffee and just get out of the house for a couple of hours.* – C9.

### Rebuilding their sense of self

The third and final overarching theme that was constructed concerned how participants were able to rebuild a positive sense of self as a result of the SP process. Two sub-themes were identified: clients reasserting positive identities about themselves and finding meaning and purpose through their groups. Clients reported these benefits, which were often missed by link workers, as the most meaningful to their experience in the SP process.

#### Reasserting a positive identity

Multiple clients noted that seeing themselves as separate from others, lacking a sense of self, and feeling dehumanised or stigmatised were core to their experience of loneliness and isolation at the beginning of their SP experience.

*Yeah, I’m really upset at the fact that I’m nothing. Then I was told the other day, “If you’ve got to fill in any of these forms, you’ve got to put in W for widow,” and it really knocked me. I was like, “Come on, I’m still just me.”* – C1.

This was often missed by link workers, who were more attuned to the barriers clients were experiencing, or specific aspects of feeling separate or stigmatised due to belonging to the client’s specific background (e.g. linguistically diverse clients). Some clients additionally described how the link workers themselves could be detrimental by focussing on barriers and activities at the expense of being treated as a whole person beyond these.

*The first one, she just looked at the sheet and fired out the dull, just straight, boring questions. The other one was a bit more interested and more attentive to what you said rather than just looking at a sheet and scribbling down a few answers… Most people have a story in life, and everyone’s got a few different stories to one another.* – C4

However, as clients joined groups they and their experiences were validated by others, and the acceptance and support they received enabled them to feel connected with others. This in turn resulted in clients redeveloping a self-concept, frequently described as feeling like a person with positive qualities and attributes:

*I guess, like I said before, it’s made me feel like I’m a human being again.* – C1.

#### Finding purpose and growth in groups

Clients and link workers frequently recounted that their experiences with loneliness and isolation were a result of major life change. This change was often characterised by a sense of loss and uncertainty about their situation. This was most commonly from the death a loved one, retirement or unemployment, health conditions and disability, or the repeated lockdowns and change during the COVID-19 pandemic. With this loss and uncertainty came a sense of purposelessness as clients struggled to find direction and create meaning in their new circumstances:

*We had a lady who had been a carer for her husband for many, many years and she was really quite healthy and fit herself, even though she was well into her 80s, and her whole life was centred around caring for her husband and she did nothing else. But when he was gone, she was really left with this void.* – LW5

However, in joining groups many clients found purpose, meaning and a sense of achievement in their activities and relationship with their members. For many clients, this was as small as having regular events and interactions to look forward to and plan for. Other clients took this further, taking on leadership or facilitator roles in their groups and finding a sense of achievement through supporting their peers.

*And I’ve got nice things happening, like people are doing arts and crafts and I’m involved in creating a market for them, because it’s for greeting cards and stuff like that that they make. It’s just that’s something that wouldn’t have happened if I hadn’t got out there and networked it a little bit and just started to participate a little bit differently.* – C7.

## Discussion

SP services are still few and far between in Australia and there is a clear need to understand the psychosocial processes through which successful SP alleviates loneliness to guide its implementation. To address this need, this study aimed to examine perspectives of both link workers and clients on how SP helps clients to develop and sustain shared social identities in community-based group programmes. Link workers and clients described experiences that fell into three broad themes about breaking down barriers to social connection, finding fit with others and motiving further change, and rebuilding the client’s sense of self through identity and meaning.

### Addressing psychosocial barriers related to social groups

Our results showed that identifying and addressing psychosocial barriers early in the s SP process was critical to its success according to both link workers and clients. The most substantial barriers related to social anxiety, sense of safety and self-confidence. These psychosocial barriers echo much of the research on factors associated with loneliness (Cacioppo and Cacioppo, 2018; Gong and Nikitin, 2021; Stuart et al., 2022; Vanhalst et al., 2017). These results indicate the importance of prioritising psychosocial barriers early in the SP process, dedicating time to identify and address the specific needs of the client before or as they join groups. If these barriers weren’t addressed, the subsequent social experiences are likely to be less positive, and the connection building would be hampered by the client’s anxiety, reduced sense of safety and lack of confidence (David et al., 2015; He, 2022).

Furthermore, clients reported that it wasn’t enough to focus on these barriers during intake to the programme. Rather, it was important the whole SP process – from the link worker to the group facilitators and their fellow group members – reduced social pressures, facilitated a sense of safety, and built their confidence steadily. This is reflective of the link worker being able to provide advocacy and a safe environment for clients, and assist the client in finding groups that offer such an environment too (Kimberlee, 2015; Stuart et al., 2022). Attending to these barriers early and ensuring their ongoing management allowed a foundation of security and confidence for the client, and facilitated other interpersonal factors embedded within the SP process such as belonging and trust (Jen et al., 2010; Stuart et al., 2022; Tanis and Postmes, 2005; Wakefield et al., 2022).

### Developing shared social identities

Both link workers and clients indicated that a sense of fit and belonging with the group was essential to the success of the linkage process. This primarily occurred through feeling welcomed into the group by the facilitator and the members, building up trust and connection with the group, and turning this into motivation for further improvements and meaningful social interaction. When clients were welcomed into the group, they became more trusting of others and motivated to engage socially. In contrast, clients became less trusting of the SP process, and less motivated to engage with it when they felt rejected or othered by groups they were linked to.

Moreover, clients reported that the members of the group they were joining had the greatest impact on the success or failure of the linkage. Clients quickly became discouraged and disengaged in a group if they didn’t have much in common with the group members or felt excluded, regardless of their interest in the activity or relationship with the group facilitator. Conversely, when the client shared experiences with the group members and felt more supported by them, they were much more motivated to keep engaging with the group. These results reiterate the importance of a sense of belonging and commonality with group members in driving shared social identities (Kellezi et al., 2019; Tarrant et al., 2020; Wakefield et al., 2022). Furthermore, by joining groups with supportive peers clients can experience a new sense of self comprising of these positive social identities they now share with others (Khan et al., 2015; Williams et al., 2019).

### Benefits of shared social identities

While the sense of fit and belonging with groups was commonly understood to be a cornerstone of the SP process, less obvious to link workers was the resulting improvements to how clients viewed themselves. Many clients reported lacking a true sense of self in their disconnection or being limited to stigmatised identities by the views of others, in turn making it harder to find personal meaning in their current circumstances. However, this sense of self shifted positively as their anxieties and fears were addressed and sense of belonging was fostered. Alongside this, the clients often found positive meaning and outlooks in their groups, activities and achievements resulting from SP. These results echo many findings applying the Social Cure framework in SP (Kellezi et al., 2019; Stuart et al., 2022; Wakefield et al., 2022; Williams et al., 2019). Additionally, these findings parallel previous work showing how social identities promote self-esteem (Jetten et al., 2015), foster positive attributions about oneself (Cruwys et al., 2015) and shift from negative or harmful behaviours, attitudes and social groups to more positive alternatives (Dingle et al., 2015; Muldoon et al., 2019).

### Implications

Holistic models of SP have sparked widespread interest due to their capacity to make healthcare more accessible in the local community and alleviate the burden on the health systems, especially primary care settings such as hospitals and GP clinics (Cruwys et al., 2018; Global Social Prescribing Alliance, 2021; Molloy et al., 2010). However, the subsequent research from this widespread interest has resulted in a diverse assortment of SP approaches, measures and methodologies (Bickerdike et al., 2017; Chatterjee et al., 2018; Ending Loneliness Together, 2021; Husk et al., 2020; Wood et al., 2021). As such, SP has largely been an atheoretical practice despite its evidence base, lacking a consistent framework to explain how the SP process results in improved health and well-being (Frostick et al., 2019). The Social Cure approach has successfully been applied separately to examining how SP improves health and well-being via social cure processes (Kellezi et al., 2019; Wakefield et al., 2022), and how groups can act as a barrier in SP via social curse processes (Sharman et al., 2022; Stuart et al., 2022).

The present study adds to this body of research by examining both the barriers clients experience to social interaction and how SP helps address these and improve health through the social cure approach. Additionally, this study contributes to the wider body of research supporting the use social approaches alongside individualised care to improve health and well-being (Haslam et al., 2018; Wakefield et al., 2019). Lastly, the present study is adds to the SP literature through its focus on psychosocial barriers to engaging with SP services, which have been frequently underestimated compared to systemic and structural barriers despite their substantial impact on clients (Hassan et al., 2023; Stuart et al., 2022; Wood et al., 2021).

## Conclusion

SP is a promising intervention, providing community-based services that aim to address physical, mental and social health concerns and issues (Global Social Prescribing Alliance, 2021), and has the potential to alleviate burden currently experienced by the health care system resulting from social disconnection (The Royal Australian College of General Practitioners and Consumers Health Forum of Australia, 2019). This study is amongst the first to examine the psychosocial barriers to joining groups experienced by SP clients, and how SP helps overcome these through shared positive social identities. The results identified common psychosocial barriers experienced by many clients coming to SP services, as well as how groups used belonging and new social identities to foster positive change. This study corroborates previous work looking at how SP improves wellbeing through social identity processes (Kellezi et al., 2019), and has promising implications for the delivery of health services in the community and the wide healthcare system.
